# Correction: Nadarajan et al. Development, Management and Utilization of a Kiwifruit (*Actinidia* spp.) In Vitro Collection: A New Zealand Perspective. *Plants* 2023, *12*, 2009

**DOI:** 10.3390/plants14111649

**Published:** 2025-05-29

**Authors:** Jayanthi Nadarajan, Azadeh Esfandiari, Liya Mathew, Jasmine Divinagracia, Claudia Wiedow, Ed Morgan

**Affiliations:** Food Industry Science Centre, The New Zealand Institute for Plant and Food Research Limited, Palmerston North 4410, New Zealand

In the original publication [1], there was a mistake in Figure 5 as published. This figure included unpublished data, which is not appropriate for a review article. This figure has been updated by removing the unpublished data. The corrected Figure 5 appears below.

There was an error in the original publication. In the last paragraph on Section 2.4, paragraph 3, a reference to Figure 5 was not made in the sentence starting with “This protocol was successfully tested …… axillary shoots”. A correction has been made to this sentence by adding (Figure 5) at the end of the sentence. The sentence beginning “We tested this protocol …… (Figure 5)” is not relevant anymore following the update of Figure 5. A correction has been made to delete this sentence. The corrected paragraph is as follows:

At PFR, a cryopreservation protocol has been developed for the long-term storage of kiwifruit using a droplet vitrification protocol [18]. This protocol was successfully tested on nine genotypes from five species, i.e., *A. chinensis* var. *chinensis*, *A. chinensis* var. *deliciosa*, *A. arguta*, *A. macrosperma* and *A. polygama*, utilizing shoot tips around 1 mm in size excised from two-week-old axillary shoots (Figure 5). These young shoots were obtained by growing nodal cuttings sourced from six-week-old in vitro grown mother plants. In our study, we observed that the age of the mother plant and size of the explant can determine the success of cryopreservation; use of younger donor plants and smaller explants significantly increasing regeneration [18]. The response of kiwifruit plants to in vitro culture is not just species-specific but also genotype-specific [32]. Hence, significant variation in post-cryopreservation regeneration between different genotypes was expected, and was observed to range from 59% to 88% [33,34]. Our results confirmed that different kiwifruit genotypes respond differently to the stresses imposed at various stages of the cryopreservation protocol [33,34]. Since post-cryopreservation regeneration is higher than the suggested baseline of 40% for cryobanks [35], we will be implementing this cryopreservation protocol for long-term kiwifruit germplasm conservation.

The authors state that the scientific conclusions are unaffected. This correction was approved by the Academic Editor. The original publication has also been updated.

## Figures and Tables

**Figure 5:**
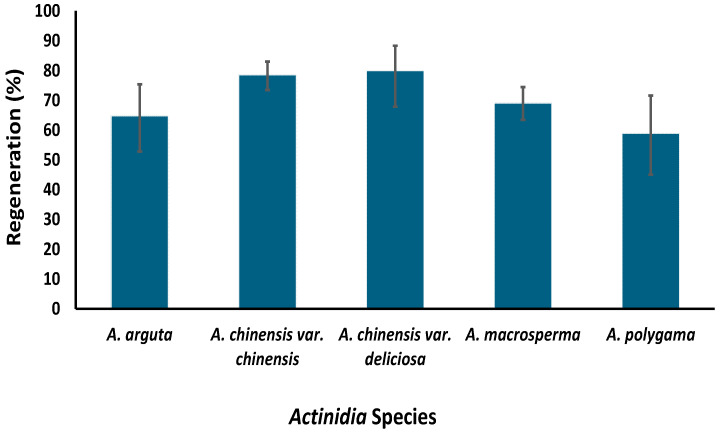
Regeneration percentages of five kiwifruit species following cryopreservation using a protocol established at The New Zealand Institute for Plant and Food Research Limited (reproduced from Table 2 [18] with permission from Springer Nature License Number 6013930561362).
