# Supplementary material for: Development, Management and Utilization of a Kiwifruit (Actinidia spp.) In Vitro Collection: A New Zealand Perspective
Source: Plants (Basel). 2023 May 17;12(10):2009. doi: 10.3390/plants12102009 (PMC10222645; doi:10.3390/plants12102009)
Supplement: Supplementary file 1 [file plants-12-02009-s001.zip › plants-2294962-supplementary.pdf]

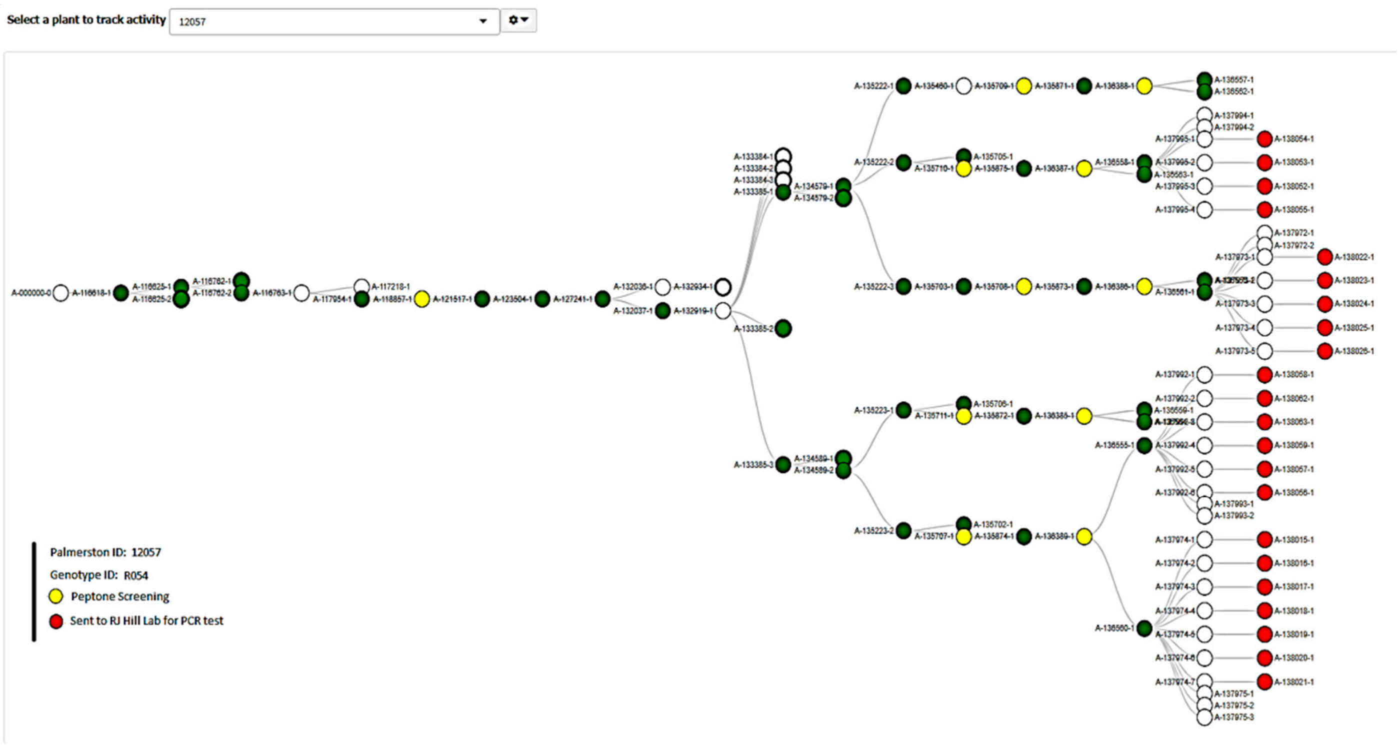

Figure S1. Example of activities recorded in GMS for one genotype. The workflow starts at initiation, with subsequent activities until being sent for testing. Each 'Activity Event ID' number (illustrated as a circle) represents one activity.
